# Numerical method investigation on the aggregation characteristics of non-spherical particles

**DOI:** 10.1371/journal.pone.0282804

**Published:** 2023-03-08

**Authors:** Pan Gao, Qikun Wang, Tangjing Liu

**Affiliations:** School of Energy and Power Engineering, University of Shanghai for Science and Technology, Shanghai, China; State University of New York at Binghamton: Binghamton University, UNITED STATES

## Abstract

Under the background of the mechanical mechanism research of microfluidic technology for separating and screening pipeline particulate matter, this paper proposes an improved relative motion model by combining the multiple reference frame method and the relative motion model. Worked with a quasi-fixed constant method, this model can numerically calculate the aggregation features of non-spherical particles in the low Reynolds number channels. The results demonstrate that when *Re* = 40~80, ellipsoids exhibit an aggregation trend similar to circular particles with the same diameter as its largest circumscribed sphere. The aggregation position is affected by the ratio of long and short axes of particles, and the distribution trend is determined by the relative size of these particles. When the channel’s Reynolds number is less than the critical Reynolds number, the aggregation position of elliptical particles will be closer to the pipe center with the increase in the Reynolds number, which is contrary to the aggregation tendency of circular particles more proximate to the pipe wall with the increase in the Reynolds number. This finding provides a novel idea and method for further exploring the aggregation rules of non-spherical particles and offers substantial guidance for separating and monitoring pipeline particulate matter via microfluidic technology and other related industrial applications.

## Introduction

Particle two-phase flow is a common flow form in nature. It also exists widely in some micro-flows with small channel sizes and velocities. Segre and Silberberg [1, 2] found that the particles uniformly dispersed in the low Reynolds number fluid in the straight pipe with a circular cross-section, after moving for a long enough distance with the fluid, would be subject to the effect of the transverse lift perpendicular to the main flow direction, resulting in the “inertial gathering” phenomenon on the ring location about 0.6 times the half diameter from the center of the pipe.

Based on this phenomenon, many related industrial engineering applications have been developed, which can be used in the field of mechanical engineering for water purification and particle screening and biochemistry separation of cells and colloidal particles, such as the separation of blood cells in microfluidic chips and the separation of active coke particles in desulfurization reactors [3, 4]. This kind of solid-liquid separation device has the characteristics of low energy consumption, large output, high efficiency, etc. Numerous applications have also promoted research on the mechanical properties of particle aggregation [5–9].

Computational fluid dynamics (CFD) can provide this information effectively and accurately and facilitate the easy identification of flow details, including the stress condition of the sphere in the flow [10–16]. Di Carlo et al. [3, 17] first proposed the “relative relativity of motion” by converting the transient flow field into a quasi-fixed flow field to simplify the calculation. Although the principle only applies to a single particle and ignores the interaction between particles, the particle aggregation phenomenon primarily exists in low-concentration particle solutions with a volume concentration of about 1%. Moreover, the computed results are consistent with the experimental results; hence, this simplification is reasonable to a certain extent. Based on this principle, a quasi-fixed constant calculation model–the relative motion model of a single spherical particle–is proposed [18]. Wang et al. [19] conducted a numerical analysis of the mechanical characteristics of spherical particles in tubes and revealed the mechanical causes of particle aggregation. They presented the hydrodynamic criteria for the stable aggregation points of particles.

Concerning particle separation, the research primarily focuses on spherical particles. Few reports concentrated on separating non-spherical and deformable particles. Furthermore, the deformable particles develop various non-spherical shapes because of the shear of the surrounding flow field during the flow process, which is the fundamental reason their aggregation characteristics differ from those of spherical particles. The examination of mechanical properties and the aggregation laws of rigid non-spherical particles of different forms is the foundation of the study of deformable particles [20]. Li et al. [21] summarized the research methods related to the motion behavior of various flowing non-spherical particles. The primary numerical methods include the immersion boundary method [22], which applies an additional volume force term to the momentum equation of the Navier-Stokes (NS) equation to fulfill the fluid-solid non-slip boundary condition, and the lattice Boltzmann method [23], which describes the motion of fluid particles from the microscopic perspective of the gas kinetics equation. Based on the lattice Boltzmann method and its similar fictitious domain method (such as DLM/FD), Huang and Lu [24] simulated the deposition process of ellipsoidal particles in the pipeline and summarized five modes of particle deposition. Hu et al. [25] stated that the inertial migration and equilibrium position of elliptical particles approached the tube wall with a decrease in the plugging ratio and the increasing ease of the length-to-diameter ratio of particles. Similarly, the equilibrium position of elliptical particles also approached the tube wall with the increase in the Reynolds number when the Reynolds number was small. Wen et al. [26] discovered that the rotation period of elliptical particles in the channel flow decreases with the reduction in the length-diameter ratio of particles. Chen et al. [27] examined the effects of particle size ratio, aspect ratio, and other parameters on the equilibrium position and velocity of single cylindrical particles under different Reynolds numbers. They concluded that the equilibrium position of particles varies with the increase in the Reynolds number when the Reynolds number of particles is greater than or less than the critical Reynolds number. Hu et al. [28] analyzed the effects of particles’ length-diameter ratio, particle concentration, fluid inertia, and clog ratio on the formation and the inertial aggregation of single lines and interlacing particle strings of elliptical particles in the channel flow, which provided excellent guidance for the corresponding study of particle sequences. However, the mathematical description of these unsteady methods is relatively complex.

Qi et al. [29] applied numerical simulation and found that with a low Reynolds number, the particles in a stable state always rotate around the shortest axis of symmetry. The long axis is parallel to the *x*–*y* plane. In this case, the shear plane is a stable rotating orbit. Yu et al. [30] and Hur et al. [31] discovered that the particles flowed periodically, and the flow patterns were categorized as “focused,” “bouncing,” and “translating,” respectively. They observed that under a specific Reynolds number, the maximum external ball diameter *D*_max_ can better identify the aggregation position of non-spherical particles than other parameters. The aggregation trend of various non-spherical particles is similar to that of *D*_max_ spherical particles, such as cylinders, disks, and doublets. Due to the translation and asymmetric rotation of irregular spherical particles flowing with the fluid, such flow fields still belong to the unsteady and dynamic boundary problem, even in the relative model. The relative motion model with quasi-stationary advantage is no longer applicable. The multi-reference frame (MRF) method is a calculation method for turbomachinery proposed by Luo et al. [32], which is extremely suitable for calculating the region’s flow field with partial rotational motion. In this method, the model is divided into two adjacent moving parts of the inertial coordinate system and the rotating coordinate system. The translation velocity is applied to the inertial coordinate system (flow field region) during calculation. And the rotation velocity is applied to the rotating coordinate system region (adjacent to the rotation field). The transient problem is approximately regarded as a steady-state problem, and the results at multiple characteristic positions are averaged to achieve results close to the instantaneous flow field.

In this paper, a quasi-fixed constant improved relative motion model for non-spherical particles is established to calculate the force of non-spherical particles. Based on verifying the model’s effectiveness, when the channel’s Reynolds number is less than the critical Reynolds number, the effects of parameters, such as the axial ratio of long and short axes, Reynolds number (*Re* number), the relative size on the aggregation characteristics of ellipsoid particles, the mechanical differences between the ellipsoid and the corresponding *D*_max_ spherical particles, are discussed. This example provides a new method and idea for calculating irregular spherical particles’ aggregation law. It offers substantial guidance for investigating the dynamics mechanism of the movement of spherical and deformable particles along with the filtration and screening of particulate microfluidic technology and other related industrial applications.

## Statement of the problem and the numerical approach

### Introduction to the relative motion model

At present, the frequently used calculation model for particle movement is the six-degree-of-freedom model based on the “dynamic grid” technology [33], which solves the six-degree-of-freedom equations in the Lagrangian migration equation of particles. Moreover, it also iterates the flow field around particles repeatedly to determine the motion trajectory and mechanical parameters of the entire aggregation process. However, the mathematical description of this unsteady calculation model is rather complicated. The calculation process is tedious and time-consuming. Based on this phenomenon, the relative motion model is proposed, ignoring the motion trajectories of particles and only calculating the forces at different positions in the channel. Moreover, the aggregation points of particles are determined by analyzing the force characteristics, which have been demonstrated in detail in the literature [19]. The model transfers the particle’s translation velocity to the tube wall by creating an inertial coordinate system. The coordinate calculation system is set in the particle’s center and translates with the particle. In this inertial coordinate system, the particle’s translation velocity is zero, and there is only a rotation velocity centered at the origin. Meanwhile, the tube wall moves in reverse with the particle’s velocity. Therefore, the flow field is transformed into a quasi-steady-state and has a simplified calculation.

### The implementation process of the improved relative motion model

**Fig 1:**
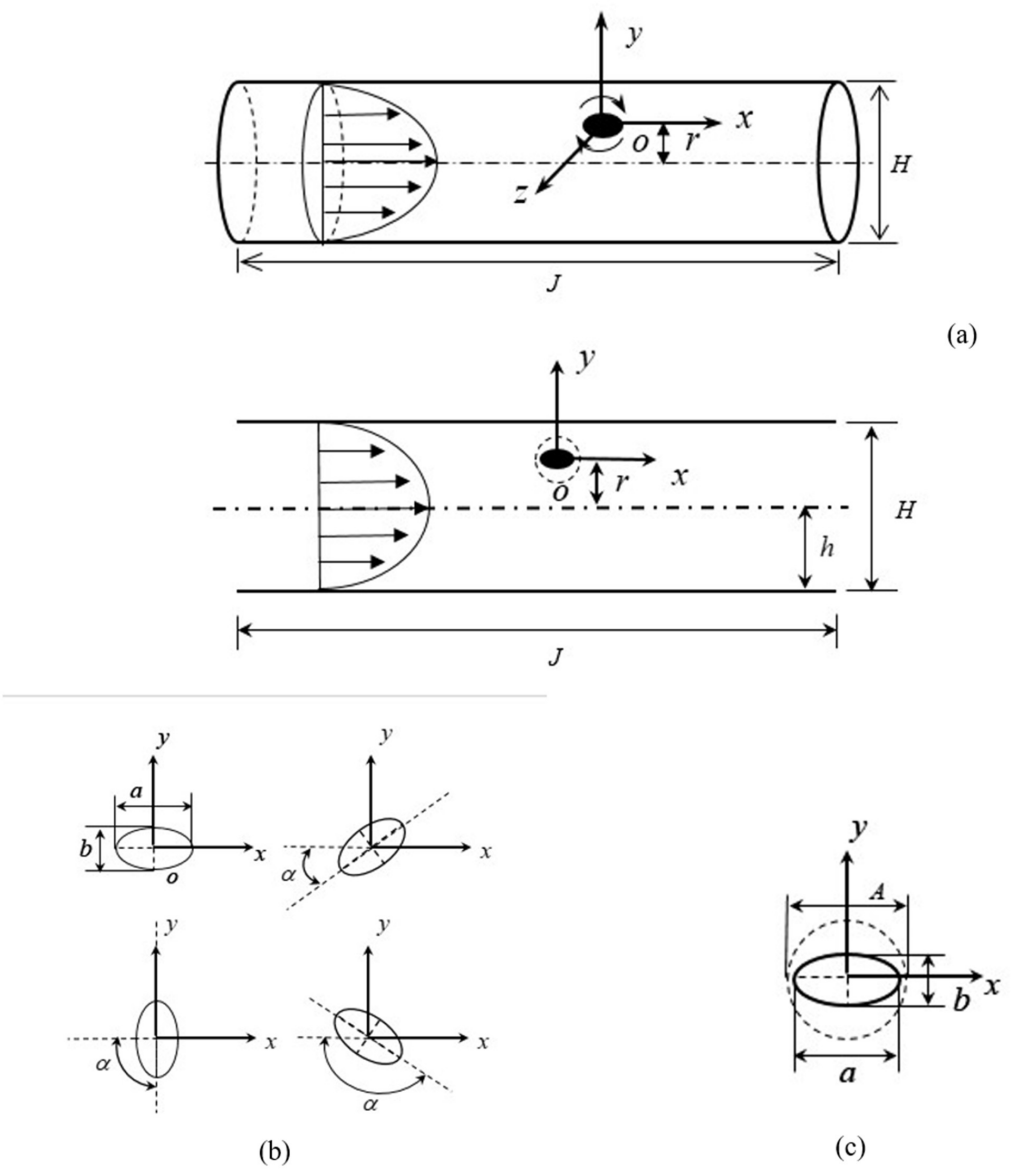
The schematics for the numerical model. (a) Schematic diagram of pipeline flow simplification, (b) rotation diagram of elliptical particles and (c) adjacent to the rotating domain particles.

Because particles in a stable state always move along the *x*–*y* shear plane, the geometrically symmetric forces in other directions can be ignored. The motion process of a single elliptical particle in a two-dimensional straight pipe Poiseuille flow is selected for analysis to simplify the calculation without losing the generality (Fig 1a). The parameters are defined as follows. The pipe diameter is defined as *H* = 2*h*; the length to avoid the effects of the entrance section is expressed as *J* = 10*H*; the long and short axes of the oval particles are *a* and b, respectively; the particle size of the dimensionless element is defined as *a*^+^ = *a*/*H*; the axis length of the dimensionless element is *L*^+^ = *a*/*b*, the long axis of the particles and the mainstream of the *x*-axis rotation angle for *α* (Fig 1b). The channel’s Reynolds number is defined as follows:

$$Re=\frac{\rho UH}{\mu }$$ (1)

where *ρ* is the fluid density, *U* is the average velocity of the fluid in the pipe, *H* is the pipe diameter, and *μ* is the dynamic viscosity of the fluid. The channel’s Reynolds number is defined by the average velocity of the fluid in the tube, reflecting the flow state of the channel as a whole.

The translational motion of the improved relative motion model proposed in this paper is consistent with that of the relative motion model, which is set through the pipe wall motion. In contrast, the rotational motion is kept in a specific rotation area by the MRF method. According to the characteristics of the calculation model, the pipeline model is divided into four parts: the inlet area, the adjacent to the rotation area (the circular area with the same center as the elliptic particle), the adjacent to the area outside the rotation area, and the exit area.

### Numerical calculation method

This paper solves the governing equation of the steady-state Navier-Stokes equation based on the finite volume method. The fluid used is pure water at 20°C with a density of ρ = 1.0×10^3^*kg*/*m*^3^ and a dynamic viscosity of *μ* = 0.001*Pa*·*s*. The grain density is the same as the fluid density. Double precision was used in the calculation, the SIMPLE algorithm was used for the coupling of pressure and velocity, the second-order format was used for the dispersion of pressure equation, and the Quadratic Upstream Interpolation for Convective Kinematics (QUICK) scheme with third-order precision was applied for the distribution of momentum equation. For boundary conditions: The ellipsoid particle is located in the middle of the computational domain, and only the *y*-axis position of the particle center is changed; The boundary condition on the left side of the computational domain is set as the velocity inlet, and that on the right side is set as the pressure outlet; The tube wall is set as the no-slip wall surface.

$$\left\{\begin{array}{l} \nabla \cdot u=0\\ \left(u\cdot \nabla \right)u=-\frac{1}{\rho }\nabla p+\upsilon \nabla^{2}u \end{array}\right.$$ (2)

where ***p*** is the fluid pressure; ***ρ*** and ***υ*** are the fluid density and kinematic viscosity, respectively (both are incompressible fluid constants); ***u*** is the velocity in the translational coordinate system.

The final constant translational velocity *U*_*p*_ and rotational velocity *ω*_*p*_ of particles at this position is achieved by the trial and error method. *U*_*p*_ is converted into boundary conditions of the pipe wall motion, and *ω*_*p*_ will be attributed in a specific rotation area. For the translational motion, the trial and error method first assumes a velocity *U*_*p*0_ for trial calculation, determines the resultant force *F*_*x*0_ of particles along the flow direction (*x* direction), and ascertains whether the magnitude order of force is zero within a particular accuracy. If it is not zero, the wall velocity shall be updated iteratively until the resultant force order of particles in the flow direction is zero, that is, the particles translate with the fluid at the same speed at this position. For rotation, the MRF method is used to keep the speed *ω*_*p*0_ in a specific rotation area. The average value of the torque at several characteristic positions in the rotation process was taken as the trial value, and its size was constantly changed until the particles rotated with zero-order torque (judging by no longer causing lift change). When the resultant force and the rotating torque of particles in the flow direction are both zero, the force of particles in the *y* direction is the lifting force.

The initial speed is calculated using theoretical prediction to improve the calculation efficiency. The initial reference value of the translation speed is $U_{p}=2U\left[1-{\left(r^{+}\right)}^{2}\right]$, and the initial reference value of the rotation speed is $\omega =\frac{2U}{R}*r^{+}$, where $r^{+}=\frac{r}{h}$ is the dimensionless radial position of the particle, *r* is the distance between the pipe center and the particle center, and *h* is the pipe radius. For *U*_*p*_ and *ω*_*p*_, the “secant method” with a superlinear convergence is used to update the subsequent iteration data, which are calculated by Eqs (3) and (4), respectively. The findings can be obtained by using this method to iterate 6–8 times, and only about 200–300 iterations are required for one attempt.

$$U_{P2}=U_{P1}-\frac{F_{x1}\times \left(U_{P1}-U_{P0}\right)}{F_{x1}-F_{x0}}$$ (3)

$$\omega_{P2}=\omega_{P1}-\frac{M_{P1}\times \left(\omega_{P1}-\omega_{P0}\right)}{M_{P1}-M_{P0}}$$ (4)

where *U*_*pn*_ and *ω*_*pn*_ are translation and rotation speeds, respectively; *F*_*xn*_ and *M*_*pn*_ are resistance and torque, respectively. The lift *F*_*y*_ and resistance *F*_*x*_ of particles are calculated according to the following equations:

$$F_{y}=j\cdot \oint_{\Sigma }n\cdot PdS$$ (5)

$$F_{x}=i\cdot \oint_{\Sigma }n\cdot PdS$$ (6)

where ***I*** and ***j*** are the unit vectors in the *x* and *y* directions, respectively; ***P*** is the stress tensor; ***n*** is the unit vector of the external normal of the particle surface; *dS* is the area element; Σ is the particle surface. The torque of particles is calculated according to the following expression:

$$M_{P}=\oint_{\Sigma }r\times \left(n\cdot P\right)dS$$ (7)

where ***r*** is the vector diameter.

## Discussion on several key parameters

### Discussion of grid independence

**Fig 2:**
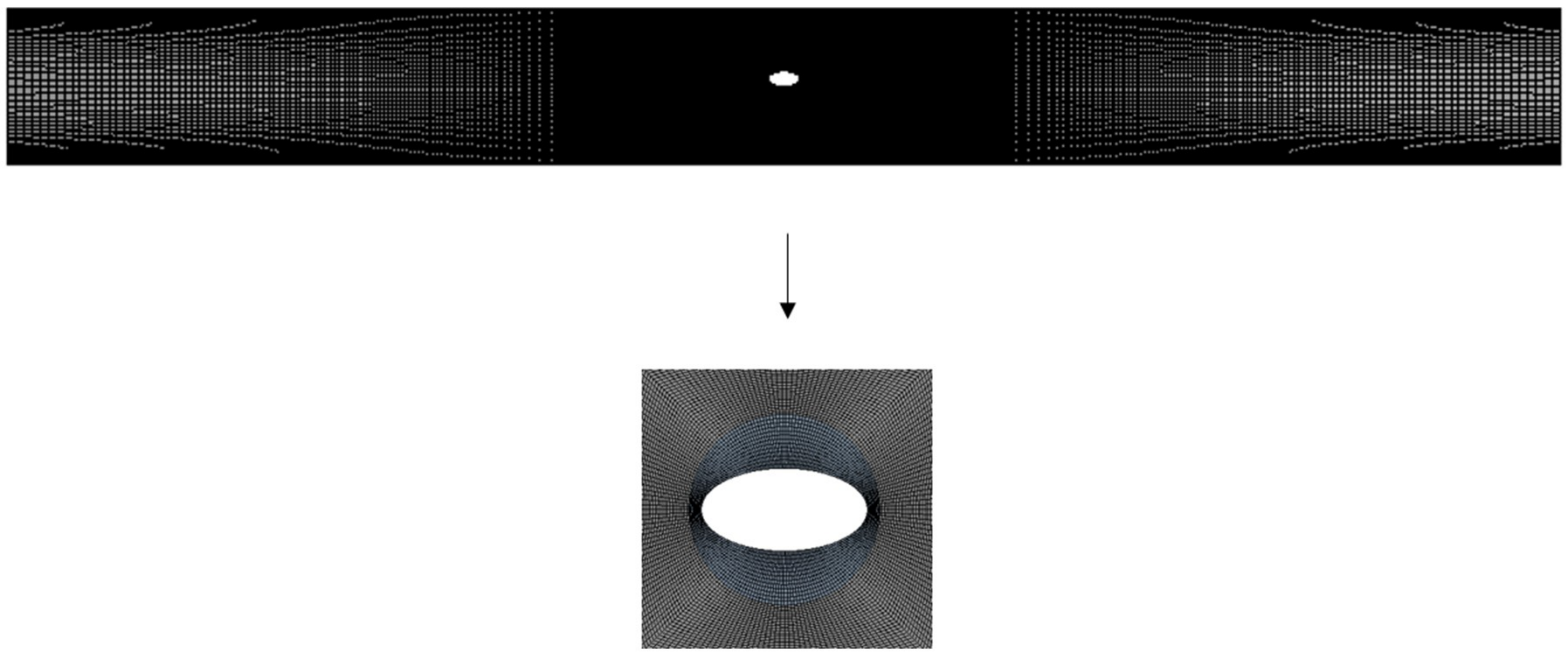
Schematic diagram of the grid.

**Fig 3:**
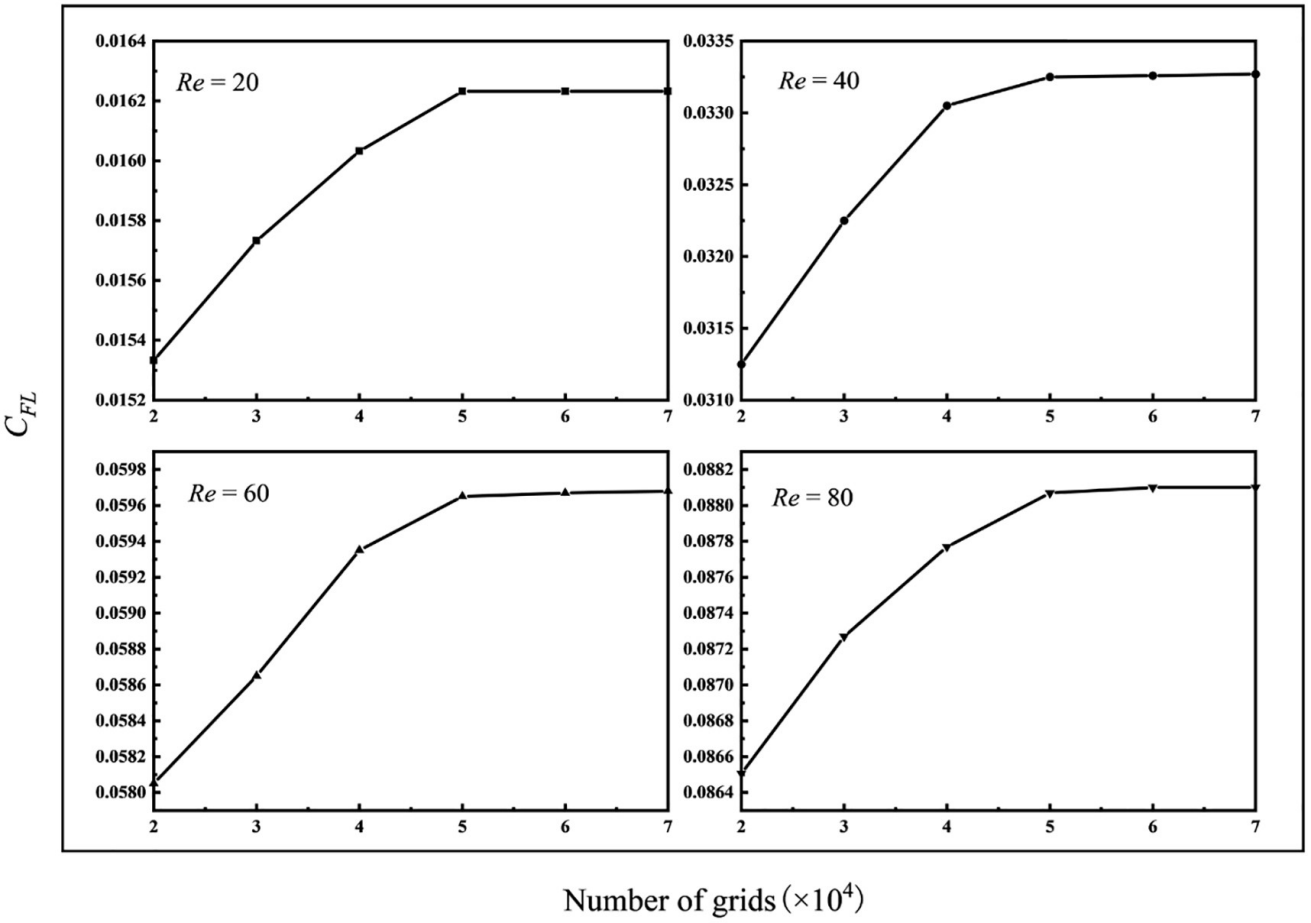
Grid independence verification.

A combination of the structured and unstructured grids is used in this paper to divide the computing domain. A structured grid is generated in the area outside the inlet and outlet and adjacent rotation domain, and an unstructured grid is generated in the particle’s adjacent rotation domain. The internal grid surface of the interface computing domain is used between the two regions, and the boundary layer grid between the fluid and the particle is encrypted (Fig 2). In order to improve the calculation accuracy, the grid independence was verified under the specific conditions (particle size *a*^+^ = 0.2, the relative radial position *r*^+^ = 0.1, and the rotation angle for the *α* = 0°). Define the lift coefficient *C*_*FL*_:

$$C_{FL}=\frac{F_{y}}{\rho U^{2}a^{4}/H^{2}}$$ (8)

where *F*_*y*_ is the lift force of the particle surface in the *y* direction, *a* is the diameter of the circular particle/the length of the long axis of the elliptical particle, and *H* is the pipe’s diameter. When the number of grids reached about 51,663, the lift coefficient of the particles remained unchanged. Considering the calculation accuracy and economy, this paper finally determines that the number of grids in the calculation domain should be controlled at about 55,000 (Fig 3).

### Determination of adjacent rotational region size

Relevant studies [34] have demonstrated that the size of the rotation domain of the MRF method is not arbitrary, depending on the flow state of the fluid around the rotation domain, which exerts a specific effect on the numerical results and should be analyzed in tandem with the computational model. Lee et al. [35] revealed that the optimal size of the inner rotating fluid region of the impeller blade of an agitator was roughly 1.5 times the height of the upper and lower blades of the impeller disk and one-sixth times the impeller diameter from the top of the impeller. Aubin et al. [36] found via the comparison of experiment and numerical calculation that in the aerated tank stirred by the axial impeller (when *Re*>30), the rotation region size of the MRF method exerts a significant impact on the results, which should be reasonably determined according to the flow conditions. Patil et al. [37] numerically investigated the flow characteristics of water in the stirred tank with full baffles and reasonably matched the calculated results according to the literature data to optimize the size of the rotating fluid region in the MRF model. It was discovered that moderate-sized inner rotating fluid region was the best. Moreover, too large or too small size would result in a negative correlation between the calculated results and the literature data.

The relative size of the adjacent rotation domain of the elliptic particle is defined as *A*^+^ = *A*/*a*; where *A* is the diameter of the rotation domain and *A* is always greater than *a* (Fig 1c). The conditions of *Re* = 40, *L*^+^ = 2, *a*^+^ = 0.1, and *r*^+^ = 0.1 were selected in this paper, and the torque of the same motion process is calculated using the dynamic grid model with specified speed along with the improved relative motion model with different adjacent rotation domain sizes *A*^+^. Taking the dynamic grid model and the improved relative motion model to calculate and compare the results of the same constant difference angle during the rotation process.

**Fig 4:**
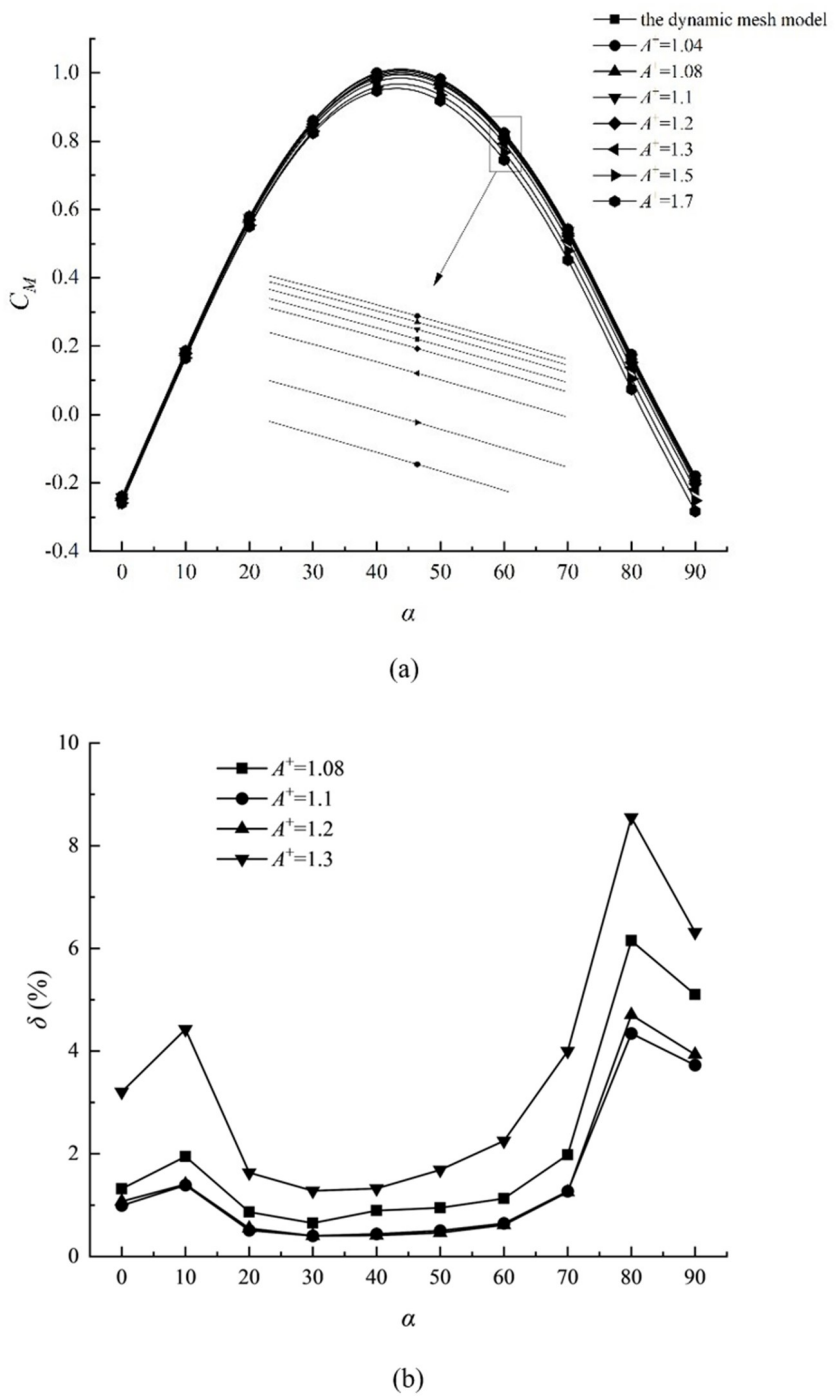
Torque distribution and relative deviation distribution under different rotation angles. (a) torque distribution and (b) relative deviation distribution.

The dimensionless torque coefficient *C*_*M*_ and relative deviation *δ* are defined as follows:

$$C_{M}=\frac{M_{P}}{\frac{1}{2}\rho U^{2}Sa}$$ (9)

$$\delta =\frac{\left|N_{1}-N_{2}\right|}{\left|N_{1}\right|}\times 100\%$$ (10)

where *N*_1_ and *N*_2_ are the standard and calculated values, respectively. Fig 4 depicts the comparison results of torque coefficients obtained by the two methods. The torque on the lower wall of the two calculation models is consistent, demonstrating a trend of increasing and decreasing. The size of the adjacent rotation domain *A*^+^ affects the torque value. When *A*^+^ is between 1.1 and 1.2, the difference between the two methods’ calculation results is less than 3%. The gap between the two methods increases when *A*^+^ decreases or increases. The selection of *A*^+^, the relative size of the adjacent rotation domain, impacts the calculation results. In the subsequent calculation, this paper selects *A*^+^ = 1.15 as the relative size of the rotation domain.

### Determination of the particle rotation interval angle

The multiple reference frame method approximates the transient problem as a steady-state problem. It needs to average the results at various characteristic positions to get them close to the instantaneous flow field. While taking multiple characteristic positions for steady-state calculation and averaging the results, too few and too many value feature positions within a 360° circle will affect the accuracy and calculation amount of the calculation results. In this case, it is necessary to determine the feature positions by defining the interval angle distance of equidistant equipartition.

**Fig 5:**
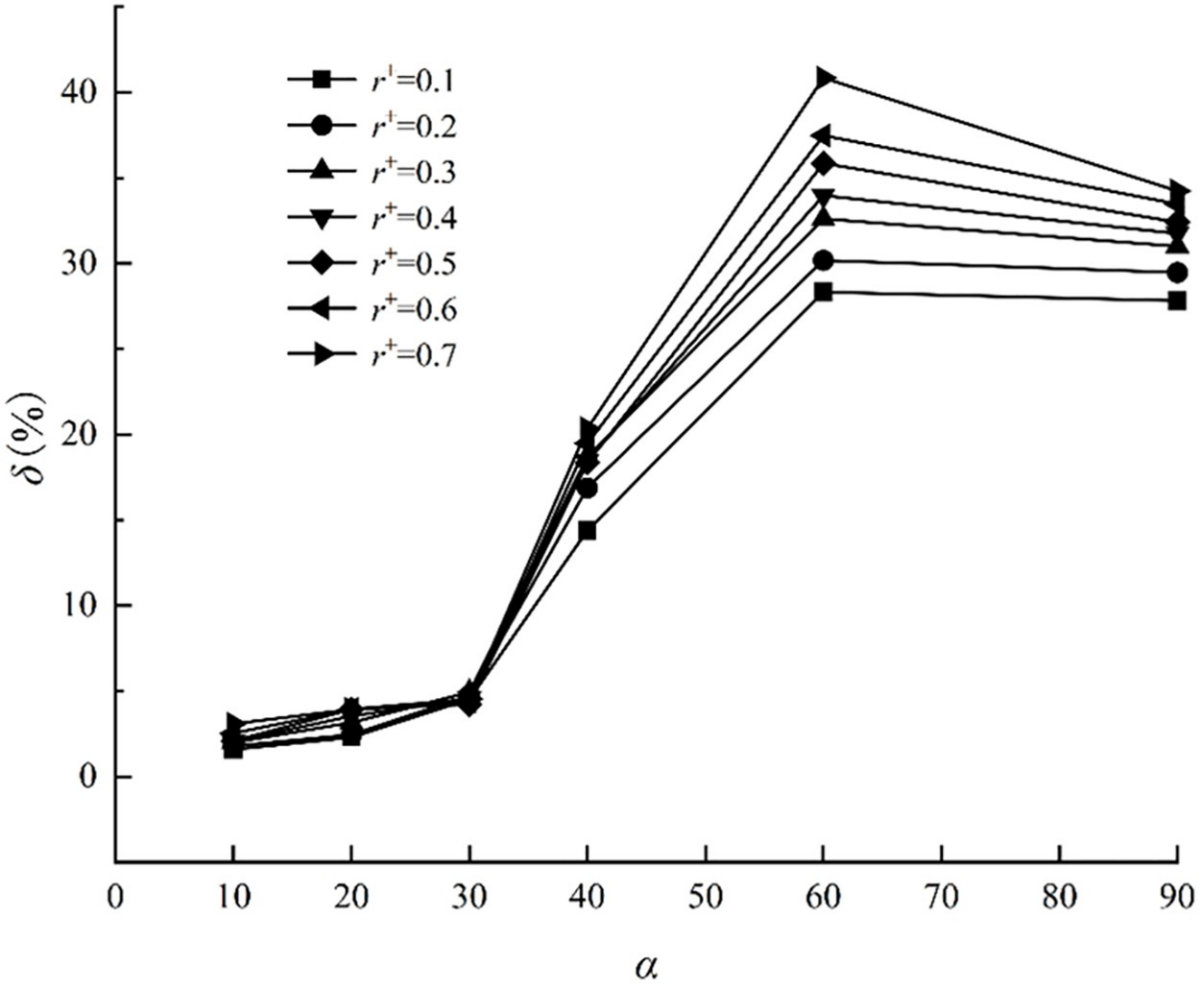
Average deviation distribution of different interval angles.

To determine the size of the interval angle, this paper uses the dynamic grid model and the improved relative motion model of the given velocity under the conditions of *Re* = 40, *a*^+^ = 0.2, and *r*^+^ = 0.1–0.7. The translation and rotation velocities are predicted using the corresponding theory of the transverse position. The characteristic position is taken as a period of 360° in every 10°–90°, which can be equally divided from each other. Fig 5 depicts the comparison between the standard value calculated by the two-degree-of-freedom model and the average value calculated by the steady model under different spacing angles. When the gap angle is large, for example, when the average angle distances of every 40°, 60°, and 90° are taken, the result is quite different from that of the dynamic grid. The relative error of the two decreases to less than 4% when the interval angle is reduced to every 30°. In the following calculation, the torque of elliptical particles is averaged at an equal interval of every 30° to consider accuracy and efficiency.

### Method validation

At low *Re*, the force calculation of regular circular particles based on the relative motion model has been well demonstrated by the literature [19]. In contrast, the improved relative motion model must be used for the comparison verification of the six-degree-of-freedom model for the force calculation of irregular spherical particles. The lift force, drag force, and torque subjected to the ellipsoidal model exhibit periodic changes due to the asymmetry of the ellipsoidal model while using the six-degree-of-freedom model to calculate the motion of the ellipsoidal particle model in a two-dimensional tube flow. When the relative deviations of lift, resistance, torque, translation velocity, and rotation angular velocity in a certain period are smaller than that in the previous period at different included angles, the flow can be determined to be in a stable periodic motion state. The lift resistance and torque values over the period are averaged at several angles. The average value is the lift force, drag force, and torque at the transverse position under *Re*.

Under the working conditions of *Re* = 40, *L*^+^ = 2 and *a*^+^ = 0.1, the improved relative motion model is used to conduct the trial calculation of the lift force of elliptical particles in different transverse positions. Meanwhile, the two-degree and six-degree-of-freedom models are used to calculate the force of particles in the above working conditions. The drag, torque, and lift force are averaged for each translation and rotation speed and the average value of the test.

**Fig 6:**
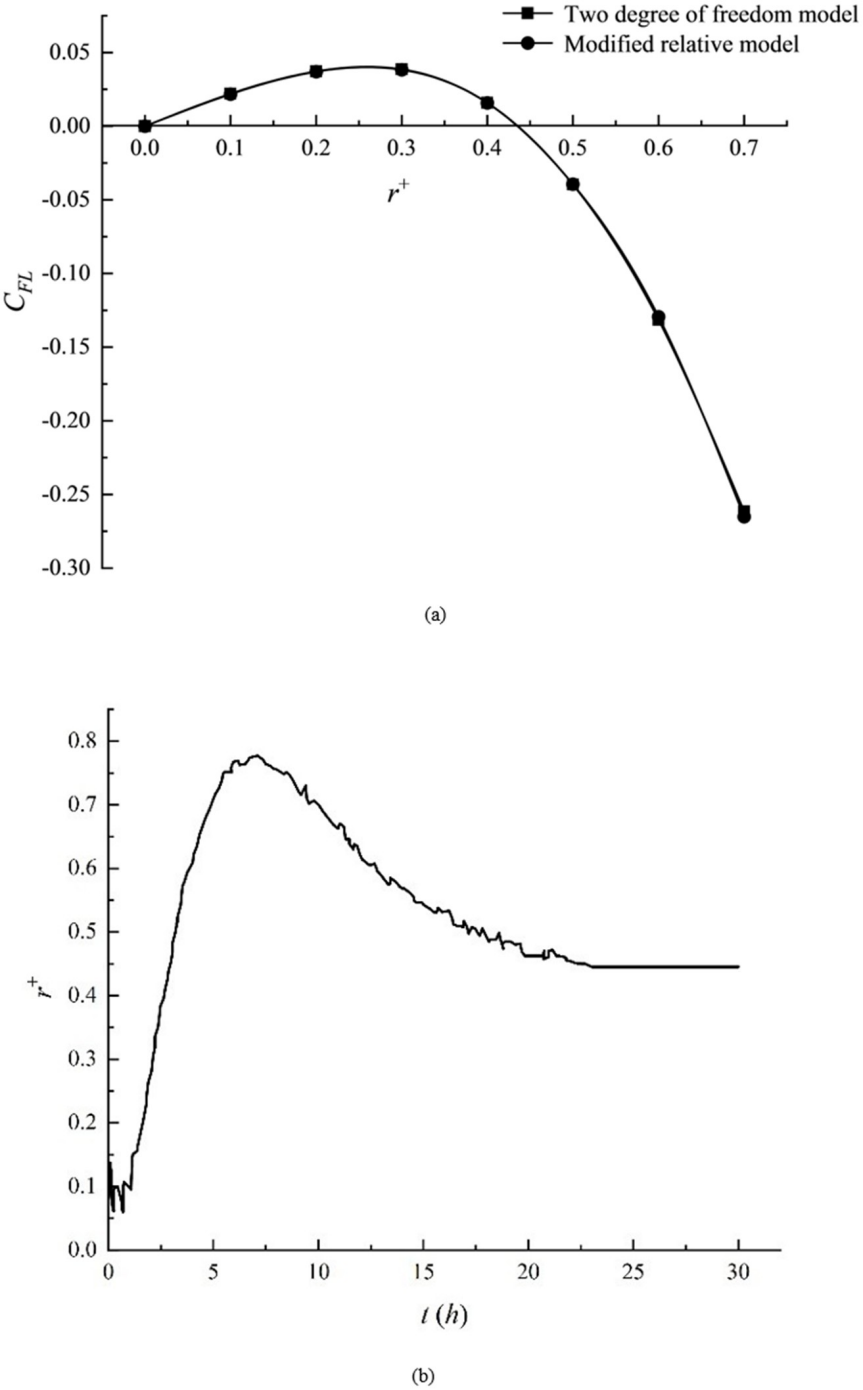
Average deviation distribution of different interval angles. (a) lift distribution comparison and (b) trajectory distribution.

Fig 6 shows that the lift distribution calculated by the improved relative motion model and the six-degree-of-freedom model is consistent. Similarly, Fig 6a demonstrates that the zero lift point is roughly located in the transverse position of *r*^+^≈0.43, which is consistent with the aggregation point of *r*^+^≈0.44 calculated by the six-degree-of-freedom model in Fig 6b, suggesting the effectiveness of the improved relative motion model in the calculation.

## Results and discussions

### Effect of flow field parameters on elliptical particle aggregation

**Fig 7:**
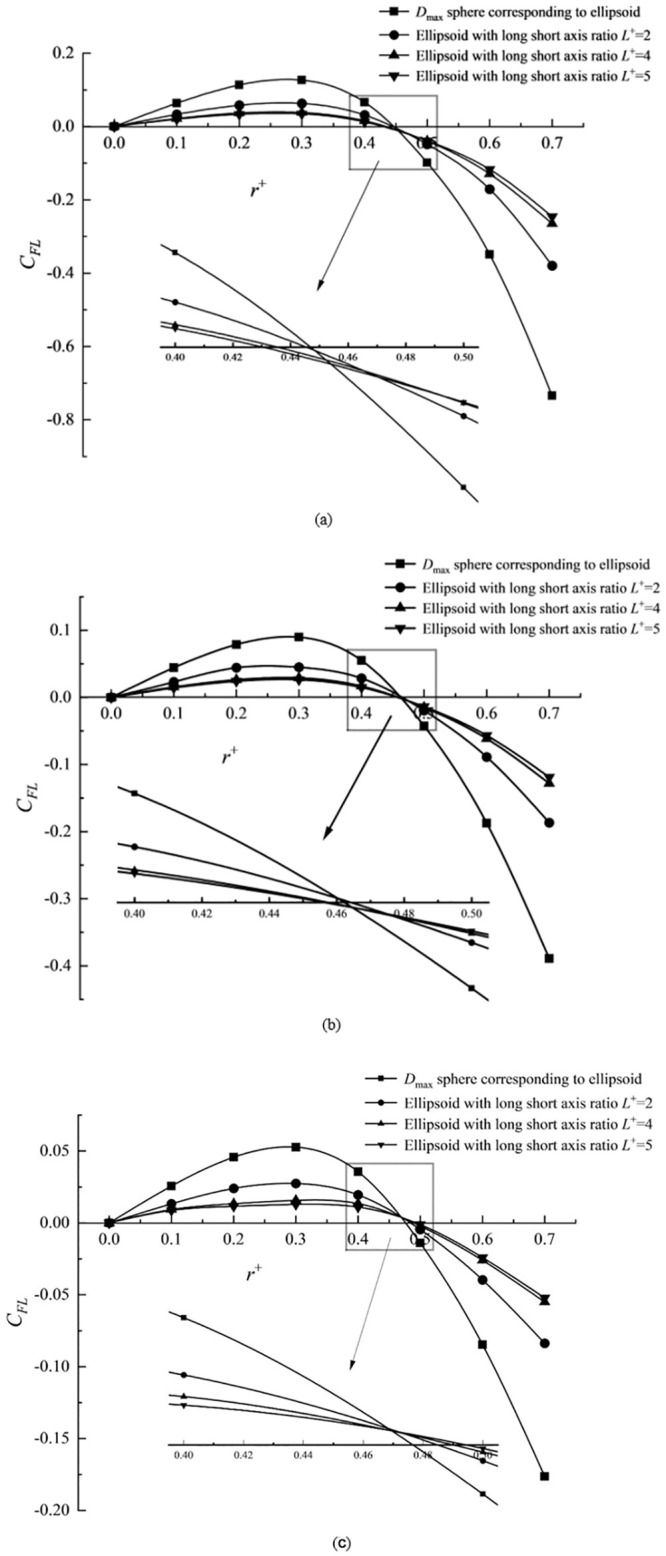
Lift distribution of elliptical particles and its corresponding circular particles under different relative size and long short axis ratio. (a) *a*^+^ = 0.2, (b) *a*^+^ = 0.15 and (c) *a*^+^ = 0.1.

Fig 7 shows the lift distribution of elliptical particles with different particle relative sizes along with long and short axes ratios *L*^+^ and their corresponding maximum diameter *D*_max_ of round particles when *Re* = 40. The lift force distribution of elliptic particles in the transverse space is consistent with that of circular particles, illustrating a parabolic distribution. The aggregation positions of elliptical particles with different axial ratios of long and short *L*^+^ are distributed near circular particles’ aggregation positions. The distribution tendency of the positions is determined by the relative size *a*^+^. When *a*^+^ is larger (e.g., *a*^+^ = 0.2), the elliptical particles are located in a minor transverse position (closer to the pipe center). When *a*^+^ is smaller (e.g., *a*^+^ = 0.1), the elliptic particles are located in a larger transverse place (closer to the tube wall). For moderate *a*^+^ (e.g., *a*^+^ = 0.15), the aggregation positions of elliptic particles are distributed on both sides of circular particles. This result is consistent with the experimental prediction results observed by Hur et al. [31] in the rectangular channel, non-circular particles have an aggregation trend similar to that of *D*_max_ circular particles, and the distribution trend is affected by the relative particle size.

The inertial lift force of a particle is essentially the projected *y* direction component of the resultant force exerted by the fluid around the particle on its surface. The forces of fluids on solid surfaces fall into two main categories: the resultant force of the pressure inertial lift force and the resultant force of shear inertia lift. With the increased distance from the pipeline axis, the inertial lift first increases in a positive direction (it points to the pipe wall). It then gradually decreases until it becomes negative (its direction changes to the direction pointing to the pipeline axis). The shear inertia lift always means to the axis of the pipeline. The shear inertia lift is minimal near the pipeline axis, almost zero. The lift will increase rapidly near the pipe wall, and all those point to the axis of the pipeline. Because of the shape rules of circular particles, the resultant force of surface forces on different transverse positions does not change with translation and rotation. The aggregation position of circular particles is relatively fixed. However, the asymmetric periodic rotation of elliptical particles will significantly lower the pressure of inertial lift force. Larger particles will have a larger negative value pointing to the pipe axis, and the resultant surface force will be greater than circular particles of the same size, making their position closer to the pipe center and vice versa.

### Comparison of the mechanical properties of particles

**Fig 8:**
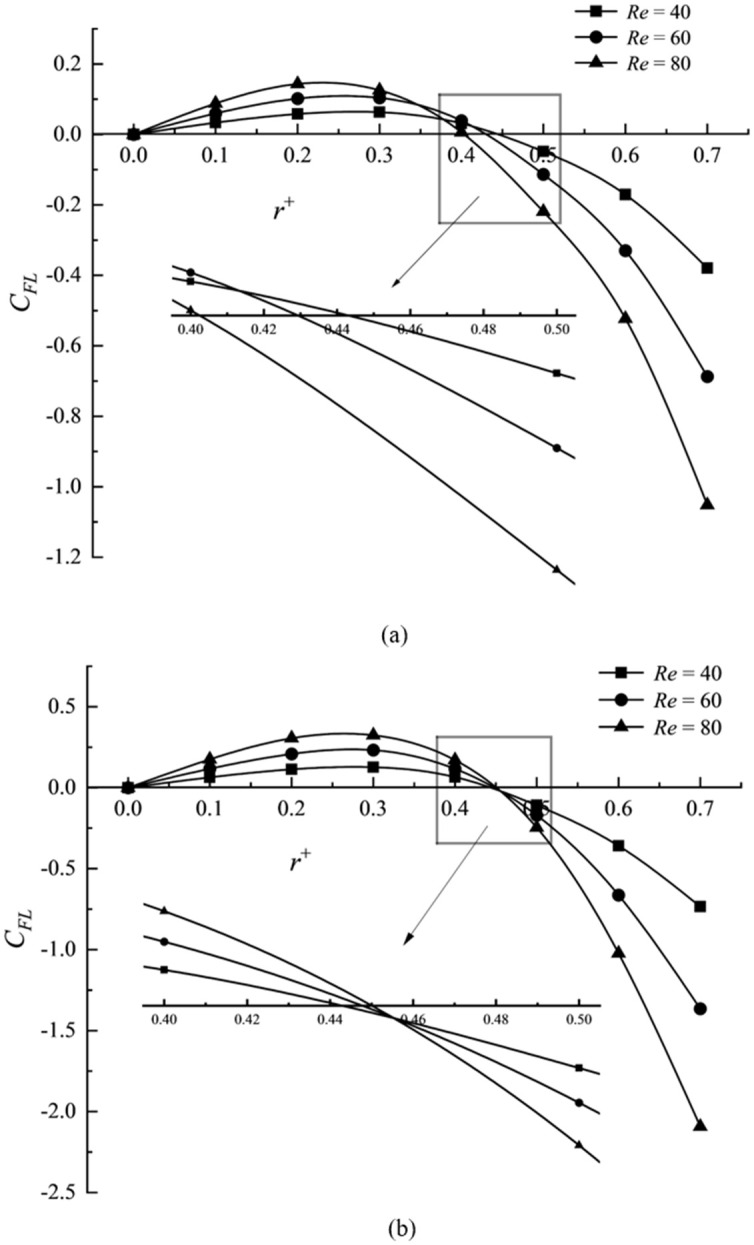
Lift distribution of elliptical particles and its corresponding circular particles under different *Re*. (a) elliptical particles and (b) circular particles.

**Fig 9:**
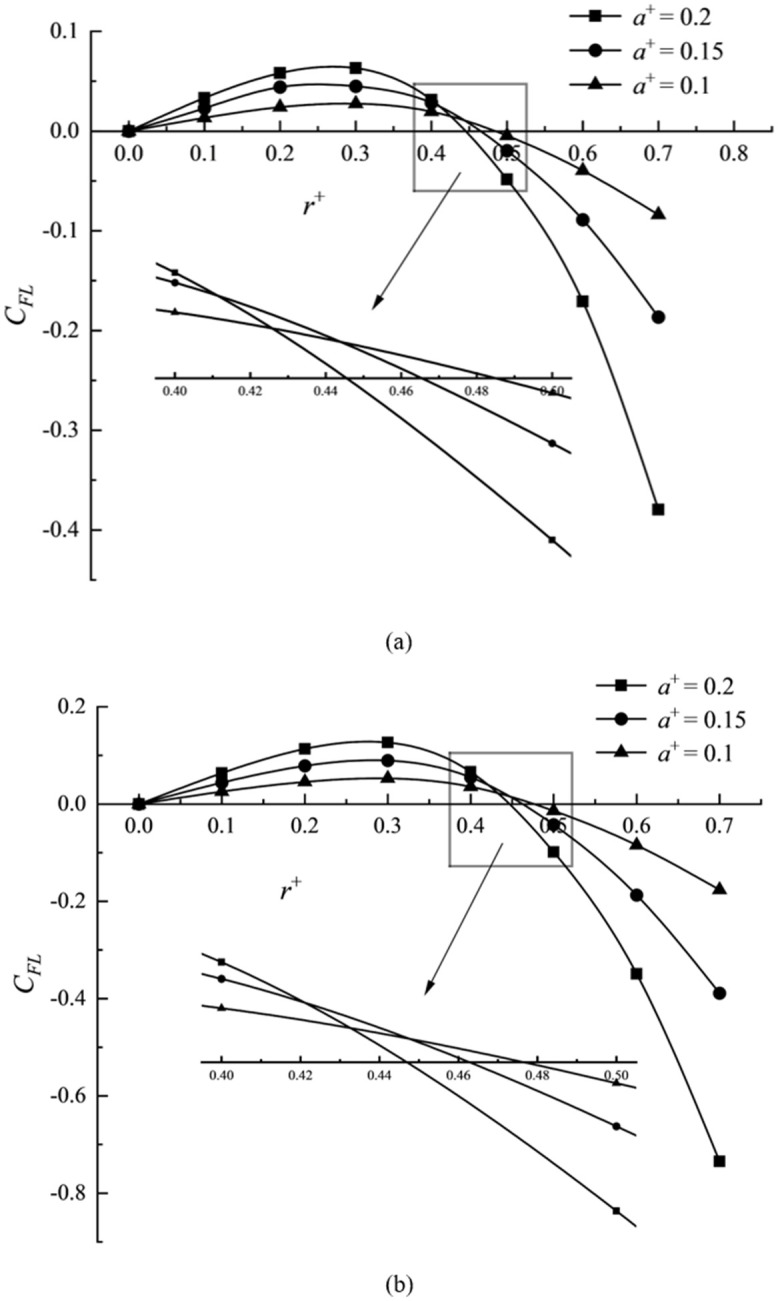
Lift distribution of elliptical particles and its corresponding circular particles under different relative size. (a) elliptical particles and (b) circular particles.

Figs 8 and 9 illustrate the lift distribution of elliptical particles and corresponding maximum diameters *D*_max_ circular particles with different values of *Re* when *a*^+^ = 0.2, *L*^+^ = 2, and *Re* = 40, *L*^+^ = 2, respectively. *Re* and *a*^+^ change particles’ lift distribution and aggregation point. Still, the *Re* number exerts more impact on the distribution characteristics of the aggregation position of elliptical particles than the relative size *a*^+^. Fig 8a shows that the aggregation position of elliptical particles is closer to the center of the pipe with an increase in the *Re* number, contrary to the trend in Fig 8b that the circular particles are closer to the pipe wall with an increase in the *Re* number. Fig 9a and 9b show that the influence of the relative size on the aggregation position distribution of elliptical particles is the same as that of corresponding circular particles and the closer to the center of the pipe with the increase in the relative size.

The *Re* number and the relative size of particles will increase the positive lift force (pointing to the pipe wall) and opposing lift force (pointing to the pipe wall) on the elliptic particles near the pipe center. The *Re* number will lead to a larger velocity normal gradient in the flow field. The shear inertia lift force is minimal near the pipe axis, almost zero, but increases rapidly near the pipe wall. In the transverse position near the zero lift point, the effect on the surface net force is more potent than that of the pressure inertia lift, resulting in the aggregation position closer to the pipe center.

### Comparative analysis of computational advantage

The six-degree-of-freedom model directly examines the specific motion history and detailed characteristics of particles from the transient point of view. It calculates the average force result of particles in a period in the form of time advance through repeated iterations. Compared with the improved relative motion model, the six-degree-of-freedom model takes longer but is more consistent with the objective physical nature. The Improved relative motion model approximates the transient problem to the steady-state problem. It averages the results of multiple characteristic positions, thus obtaining the results closer to the transient flow field. The aggregation position of particles is determined by the zero-point position of the lift force. The specific motion process of particles is ignored, and only the spatial distribution characteristics of the inertial lift force of stationary particles are concerned. By comprehensively comparing and evaluating the calculation process and results of the above two calculation models for 2D ellipsoid particles under the working conditions of *Re* = 40, *L*^+^ = 2, and *a*^+^ = 0.1, and quantifying the calculation advantages of the improved relative motion model (the specific data are shown in Table 1), the following conclusions are drawn:

1. Grid generation. In the calculation process of the six-degree-of-freedom model, because particles are in motion, their relative positions change with time. The calculated flow field is unsteady and moving boundary flow. Moreover, the “moving grid” technology should be introduced into the six-degree-of-freedom model. In the calculation, the grid must be reconstructed constantly, and it is easier to show isolated elements, causing the non-convergence of the calculation. However, because the particles are stationary for the improved relative motion model, it is only necessary to use the MRF method to generate several sets of fixed structured grids from several characteristic positions during the rotation process. The average value of the surface at the characteristic position is taken as the trial value to the average, and then the calculation can be conducted. In addition, the MRF method only needs to determine the size of the adjacent rotation region once, which can be used as the determining parameter for the next calculation without any adjustment.
2. The number of grids. For the calculation of the same working condition, the six-degree-of-freedom model requires a very long pipeline model length to ensure that the particles can reach a stable state of periodic motion, increasing the number and difficulty of the calculation grid virtually. In the above example, about 600,000 grids must be built when the six-degree-of-freedom model is used to calculate the two-dimensional particle model. Using the two-degree-of-freedom model, about 200,000 meshes need to be built to calculate each transverse position. For the improved relative motion model, because the particles are fixed, the length of the pipeline model and the number of grid layouts can be significantly reduced by placing the particles in different transverse positions of the channel, using the combination of structured and unstructured grids to generate fixed grids and only local encryption on the surface of the particles and the area near the adjacent rotation domain. After grid independence verification, each set of fixed grids only needs 50,000–60,000 grids to be calculated.
3. Calculation time. In this paper, commercial 80-core parallel machine workstations are used in the actual calculation process. Taking the above example as a reference, when the six-degree-of-freedom model is used to calculate the transverse lift force of 2D irregular spherical particles, the time step of the transient algorithm is 0.01*s* and the internal iteration times are 50–100 to ensure that each time step is iteration convergent and grid reconstruction is conducted. The total time step is about 4*s*, the total number of iterations is about 10,000–20,000, and the required time is about 1–2 days. Suppose the improved relative motion model is adopted. In that case, only six sets of fixed grids must be determined according to the six characteristic positions of the rotation process in each transverse position according to the MRF method. The trial speed value is brought into each set of fixed grids for calculation. The lift resistance of all models is obtained and averaged, and the next trial speed value is obtained by trial and error. The above method is used for eight to ten trials. Each trial must calculate six sets of fixed grids. The number of iterations of a single fixed grid is only 150–200 to determine the lift resistance. A single trial only takes 20–30 minutes, and the whole trial process only takes 3–5 hours.

**Table 1:** Comparison of calculation process and results of two calculation models.

*Project*	The six-degree-of-freedom model	The improved relative motion model
Number of grids	600,000	55,000
Total iterations number	18300	158
Iteration time(*s*)	1–2 days	3–5 hours

In summary, the improved relative motion model is simpler and more economical than the six-degree-of-freedom model.

## Conclusions

1. At a low *Re* number, the lift distribution of elliptical particles in the radial space is consistent with that of circular particles, showing a parabolic distribution. The elliptical particles with different axial ratios of length exhibit an aggregation trend similar to that of circular particles with the same maximum diameter of the external ball. The relative size of particles predominantly affects the aggregation trend. When the size is larger, the elliptical particles are closer to the center of the pipe than the circular particles. When there is a smaller size, the elliptical particles are closer to the tube wall than the circular ones.
2. The effect of the relative size on the aggregation location distribution of elliptic particles is consistent with that of the corresponding circular particles. With the increase in particle size, the aggregation location is closer to the center of the pipeline. The effect of the *Re* number on the aggregation location distribution of elliptic particles is opposite to that of the corresponding circular particles. When the channel’s Reynolds number is less than the critical Reynolds number, the aggregation location of the circular particles is closer to the pipe wall with the increase of the *Re* number. In contrast, the elliptic particles are closer to the pipe center.

The above results demonstrate that the improved relative motion model is suitable for the calculation of the mechanical properties and aggregation law of elliptic particles in the low *Re* number channel, providing a new idea for further exploring the aggregation law of non-spherical particles and helping to reveal the dynamic mechanism of irregular spherical and deformable particles. Therefore, it can offer substantial guidance for the filtration and screening pipeline particulate matter microfluidic technology and other industrial applications.

## Supporting information

S1 Fig Large rotation area range torque distribution and relative deviation distribution under different rotation angles. Taking the dynamic grid model and the improved relative motion model to calculate and compare the results of the same constant difference angle during the rotation process, (a) torque distribution and (b) relative deviation distribution.
(DOCX)

S1 Table Average torque values of the two calculation models at different interval angles. The comparison between the standard value calculated by the two-degree-of-freedom model and the average value calculated by the steady model under different spacing angles.
(DOCX)
