# Supplementary material for: Numerical method investigation on the aggregation characteristics of non-spherical particles
Source: PLoS One. 2023 Mar 8;18(3):e0282804. doi: 10.1371/journal.pone.0282804 (PMC9994714; doi:10.1371/journal.pone.0282804)
Supplement: S1 Fig — Taking the dynamic grid model and the improved relative motion model to calculate and compare the results of the same constant difference angle during the rotation process, (a) torque distribution and (b) relative deviation distribution. (DOCX) [file pone.0282804.s001.docx]

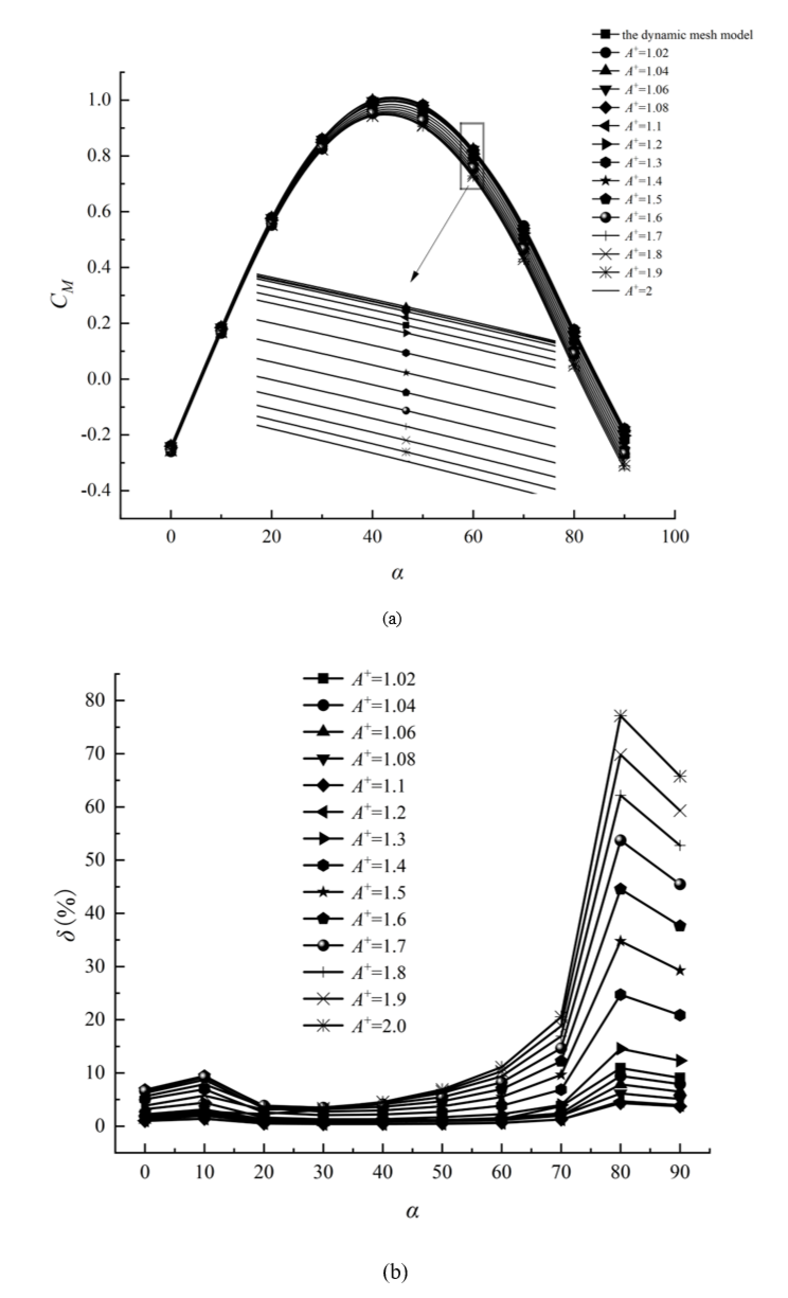


[**S1 Fig.**](https://journals.plos.org/plosone/article/file?type=supplementary&id=10.1371/journal.pone.0270918.s001) **Large rotation area range torque distribution and relative deviation distribution under different rotation angles.** Taking the dynamic grid model and the improved relative motion model to calculate and compare the results of the same constant difference angle during the rotation process, (a) torque distribution and (b) relative deviation distribution.
