# Supplementary material for: Numerical method investigation on the aggregation characteristics of non-spherical particles
Source: PLoS One. 2023 Mar 8;18(3):e0282804. doi: 10.1371/journal.pone.0282804 (PMC9994714; doi:10.1371/journal.pone.0282804)
Supplement: S1 Table — The comparison between the standard value calculated by the two-degree-of-freedom model and the average value calculated by the steady model under different spacing angles. (DOCX) [file pone.0282804.s002.docx]

### [S1 Table.](https://journals.plos.org/plosone/article/file?type=supplementary&id=10.1371/journal.pone.0270918.s002)Average torque values of the two calculation models at different interval angles. The comparison between the standard value calculated by the two-degree-of-freedom model and the average value calculated by the steady model under different spacing angles.

| transverse position | 2-dof model | 10° | 20° | 30° | 40° | 60° | 90° |
| --- | --- | --- | --- | --- | --- | --- | --- |
| *r*^+^=0.1 | -0.0086 | -0.0084 | -0.0032 | 0.0076 | 0.0128 | 0.0198 | 0.0185 |
| *r*^+^=0.2 | -0.0053 | -0.0054 | 0.0012 | 0.0087 | 0.0154 | 0.0237 | 0.0202 |
| *r*^+^=0.3 | -0.0019 | -0.0019 | 0.0064 | 0.0096 | 0.0176 | 0.0251 | 0.0235 |
| *r*^+^=0.4 | -0.0067 | -0.0068 | 0.0031 | 0.0099 | 0.0099 | 0.0323 | 0.0302 |
| *r*^+^=0.5 | -0.0143 | -0.0142 | -0.0087 | -0.0034 | 0.0053 | 0.0109 | 0.0099 |
| *r*^+^=0.6 | -0.0096 | -0.0098 | 0.0038 | 0.0169 | 0.0246 | 0.0481 | 0.0424 |
| *r*^+^=0.7 | -0.0107 | -0.0109 | 0.0104 | 0.0295 | 0.0423 | 0.0779 | 0.0725 |
